# Interventions to Influence Consulting and Antibiotic Use for Acute Respiratory Tract Infections in Children: A Systematic Review and Meta-Analysis

**DOI:** 10.1371/journal.pone.0030334

**Published:** 2012-01-27

**Authors:** Talley Andrews, Matthew Thompson, David I. Buckley, Carl Heneghan, Rick Deyo, Niamh Redmond, Patricia J. Lucas, Peter S. Blair, Alastair D. Hay

**Affiliations:** 1 Department of Family Medicine, Oregon Health and Science University, Portland, Oregon, United States of America; 2 Department of Primary Care Health Sciences, University of Oxford, Oxford, United Kingdom; 3 Academic Unit of Primary Health Care, School of Social and Community Medicine, University of Bristol, Bristol, United Kingdom; 4 School for Policy Studies, University of Bristol, Bristol, United Kingdom; 5 School of Social and Community Medicine, St Michael's Hospital, University of Bristol, Bristol, United Kingdom; University of Maryland, School of Medicine, United States of America

## Abstract

**Background:**

Respiratory tract infections (RTIs) are common in children and generally self-limiting, yet often result in consultations to primary care. Frequent consultations divert resources from care for potentially more serious conditions and increase the opportunity for antibiotic overuse. Overuse of antibiotics is associated with adverse effects and antimicrobial resistance, and has been shown to influence how patients seek care in ensuing illness episodes.

**Methodology/Principal Findings:**

We conducted a systematic review and meta-analysis to assess the effectiveness of interventions directed towards parents or caregivers which were designed to influence consulting and antibiotic use for respiratory tract infections (RTIs) in children in primary care. Main outcomes were parental consulting rate, parental knowledge, and proportion of children subsequently consuming antibiotics. Of 5,714 references, 23 studies (representing 20 interventions) met inclusion criteria. Materials designed to engage children in addition to parents were effective in modifying parental knowledge and behaviour, resulting in reductions in consulting rates ranging from 13 to 40%. Providing parents with delayed prescriptions significantly decreased reported antibiotic use (Risk Ratio (RR) 0.46 (0.40, 0.54); moreover, a delayed or no prescribing approach did not diminish parental satisfaction.

**Conclusions:**

In order to be most effective, interventions to influence parental consulting and antibiotic use should: engage children, occur prior to an illness episode, employ delayed prescribing, and provide guidance on specific symptoms. These results support the wider implementation of interventions to reduce inappropriate antibiotic use in children.

## Introduction

Respiratory tract infections (RTIs) are common in children and drive the majority of antibiotic prescribing for this population [1]. On average, a third of all children in the United Kingdom and United States are seen in primary care for RTIs or related symptoms each year [2], [3]. When parental time off work is added to the costs of health care, RTIs pose a major financial burden [1], [2], [3], [4]. Clinical uncertainty regarding the diagnosis and management of RTIs is illustrated by wide variations in antibiotic use in primary care between individual clinicians, general practitioner (GP) practices, and countries [5], [6], [7], [8]. Antibiotics can cause side effects in children, such as rash or diarrhoea, and rarely allergic reactions [9]. Overuse of antibiotics in primary care contributes to resistance [10], thus reducing the benefits of antibiotics, and can lead to subsequent “medicalisation of illness” where patients believe they need to consult when similar symptoms recur [11] – thereby creating a ‘vicious cycle’. Combined with a slowing in the development of new antibiotics, resistance constitutes a major threat to public health [12]. Although public education campaigns are ongoing in many countries [13], targeted efforts are also needed at the practice and patient level to reduce population-wide risk of antibiotic resistance.

In the UK, the Department of Health Standing Medical Advisory Committee's ‘Path of Least Resistance’ report (1998) outlined the pivotal role primary care must play to avert the public health disaster of ineffective antibiotics for serious infections [14]. Recent guidelines highlight the need for patients and primary care professionals to stop seeing a role for antibiotics in the symptomatic relief of RTIs, and to adopt “no or delayed antibiotic” prescribing for the majority of patients [15]. To apply these recommendations, knowledge translation strategies are needed at the parental level to influence consulting behaviour and use of antibiotics, and at the primary care interface to influence consultation skills and prescribing behaviour.

Our goal was to systematically review the evidence for the effectiveness of interventions directed towards parents and/or caregivers to promote more appropriate consulting and antibiotic use for children with RTIs. We originally intended to also include interventions targeted to clinicians designed to change antibiotic prescribing, but decided to focus on interventions targeted to parents and caregivers based on feedback during peer review as research in this area had not been synthesised previously. The systematic review was based on a conceptual model (**Figure S1**) developed by the research team (consisting of qualitative and quantitative researchers, primary care clinicians, and parents) that incorporates knowledge, beliefs, and attitudes regarding decisions to consult and to use antibiotics for RTIs. These factors are often informed by past experience; for example, receiving antibiotics for a previous cough or cold may reinforce the belief that antibiotics are indicated and the decision to consult [16]. As such, repeated consultation and antibiotic prescribing experiences can contribute to ‘vicious’ or ‘virtuous’ cycles.

Our rationale for adopting a comprehensive approach to interventions rather than focussing more narrowly on individual components (e.g. delayed prescribing) takes account of the relatedness of the interventions and their effects at multiple points within the parent-doctor interaction. We therefore identified interventions that are applicable to multiple components of the parent-doctor interaction surrounding consultations, including parental knowledge of when to consult, and antibiotic use (measured by parental report of filling prescriptions or giving antibiotics to their child), including parental knowledge and attitudes related to use of antibiotics.

## Methods

### Search

We searched MEDLINE/PubMed [1966–November 2011], EMBASE [1974–March 2011], CINAHL [1981–March 2011], PsycINFO, and the Cochrane Library using a combination of terms on respiratory tract infection, children and parents, education, antibiotic prescription, and consultation (**Table S1**). No limits were applied for language. One author (TA) independently screened titles and abstracts based on the inclusion criteria to identify potentially relevant studies. Following the initial search, TA reviewed reference lists of selected studies and searched related citations to identify additional references. Two authors (TA and MT) reviewed the full-text of all potentially relevant studies to determine final inclusion. Disagreements were settled through discussions with a third author (CH or DB). Review protocol is available by request.

### Selection

We included studies that used randomised, cluster randomised, or non-randomised controlled designs, or one group pre/post-test designs, to assess the effect of educational or behavioural interventions directed at caregivers to influence consulting or antibiotic use for acute RTIs in children (birth to 18 years), in developed countries (based on OECD member classification [17]). Comparisons included no-treatment or alternate treatment controls. We excluded studies that did not report outcomes for children with RTIs; study designs without a control group; evaluations of national treatment guidelines, public health interventions targeting multiple stakeholders at the population level, or diagnostic tests; studies of hospitalised children or those with serious comorbidities (e.g. cystic fibrosis, cancer, or other causes of immunocompromise); or studies from less developed countries (as the generalizability of the data from ‘less developed’ countries where the risk of severe complications from infection is higher will be limited).

### Study characteristics

We sought data on three primary outcomes related to RTIs in children: 1) parental knowledge related to consultations or parental consultation rate, 2) parental knowledge or attitudes related to antibiotic use, and 3) antibiotic use. Secondary outcomes included adverse effects, health outcomes, and costs of interventions.

### Data abstraction

Two reviewers (TA, MT) independently extracted data from included studies, using a predetermined extraction form, for study design, setting, patient population, definitions of included illnesses, intervention and comparison, theoretical framework, outcome(s) assessed, and method of assessment. Disagreements were resolved by discussion with a third author (DB or CH). When necessary we contacted study authors for additional information. Reviewers were not blinded to any aspect of the studies.

### Validity assessment

Two review authors (TA and MT) independently assessed study quality using a framework adapted from the Cochrane Handbook risk of bias tool [18]. Our framework included a set of criteria selected to assess educational and behavioural interventions. For randomized or cluster randomized trials we assessed risk of bias based on the following criteria: randomization (description of method, differences between experimental groups), blinding, description of intervention (content and mode of delivery), exposure to intervention (and consistency in delivery), and generalisability (to primary care practice). Non-randomized controlled trials were assessed on the basis of comparability of groups, intervention description, exposure to intervention, and generalisability. One-group designs were assessed on the basis of intervention description, exposure to intervention, and generalisability. A judgment of “low”, “high”, or “unclear” was made regarding the risk of bias for each criterion; based on this, each study was then given an overall judgement of “minimum”, “likely”, or “high” risk of bias (**Table S2**). The overall quality assessments were used to interpret the main findings.

### Quantitative data synthesis

For interventions which measured changes in mean or median numbers we calculated mean differences with 95% confidence intervals (CI), and for changes in rates we calculated odds ratios (OR) with 95% CI, using Yates's correction and Fisher's exact test where an expected cell was below five, for each of the study outcomes (EpiInfo version 3.4.3). Where raw data were unavailable, we presented proportional or mean differences. When possible we pooled outcomes and calculated risk ratio using a random effects model; we then calculated heterogeneity using I^2^ and where it was greater than 50% looked for clinical and statistical explanations. Considerable statistical and clinical heterogeneity prevented pooling of most outcomes; therefore results of each study are presented individually and interpreted using narrative analysis.

## Results

### Flow of included studies

From 5,714 initial studies, 137 met the criteria for full-text review, of which 114 were excluded (**Figure S2**). A total of 23 studies (representing 20 interventions) were included in the review; 12 interventions were from the US, six from the UK, and two from Israel. (Characteristics of included studies are reported in **Table S3**).

### 1. Interventions to influence parental consulting

We identified nine studies of eight interventions which aimed to change the number or rate of parental consultations for paediatric RTIs, involving a total of 1488 parents, 1580 families, and 558 children [19], [20], [21], [22], [23], [24], [25], [26], [27]. Interventions were delivered at home in four studies [22], [24], [26], [27], and at GP surgeries or emergency departments in the remainder. All interventions involved written material (e.g. book, pamphlet), complemented by brief verbal education in five studies [19], [20], [21], [24], [25]. In three of these studies, intervention materials included cartoons and/or illustrations [22], [25], [27]. The studies measured change in rate or number of consultations or re-consultations for RTIs [19], [21], [22], [25], [26], [27], or change in knowledge about reasons to consult [19], [20], [21], [23], [24], Follow-up periods ranged from 3 days to 17 months. Results are shown in **Table 1** and **2**.

**Table 1:** Effectiveness of interventions to influence parent knowledge related to consulting for respiratory tract infections in children.

Study	Age	Outcome	Intervention	Control	OR	NNT	Mean	Significance	Risk of
					[95%		difference		bias
					CI]				
Francis*	6 mo-	% “intends to consult	133/246	201/263	0.36	5		<0.001	Min.
	4 yr	if their child has	(54%)	(76.4%)	[0.24–				
		similar illness in			0.54]				
		future”							
Herman**	<18 yr	% would visit GP or							Likely
		ED for:							
		earache	40/61	101/113	0.24	4	-	<0.001	
			(66%)	(89%)	[0.09–				
					0.54]				
		cough	20/61	73/113	0.27	3	-	<0.001	
			(31%)	(64%)	[0.13–				
					0.54]				
Isaacman***	<3 yr	Mean knowledge							Likely
		score of:							
		how to administer	Verbal: 97.6	92.7	-	-	4.9	*NS*	
		medication	Written +						
			verbal: 96.9		-	-	4.2	*NS*	
		signs of symptom	Verbal: 60	44	-	-	16	*<0.05 (vs.*	
		improvement	Written +					*C)*	
			verbal: 73.2		-	-	29.2	*<0.05 (vs.*	
								*both)*	
		signs to reconsult	Verbal: 38.7	22.4	-	-	16.3	*<0.05 (vs.*	
			Written +					*C)*	
			verbal: 44.4		-	-	22.4	*<0.05 (vs.*	
								*C)*	
Morrell,	0–4 yr	% with correct							Likely
Anderson		responses for:							
		symptom							
		management							
		cough	15/51	11/47	1.36	17	-	0.66	
			(29.4%)	(23.4%)	[0.50–				
					3.71]				
		runny nose	5/51	2/47	2.45	18	-	0.44¶	
			(9.8%)	(4.3%)	[0.39–				
					19.31]				
		sore throat	13/51	10/47	1.27	24	-	0.8	
			(25.5%)	(21.3%)	[0.45–				
					3.59]				
Robbins	<6 mo	% know when to							Likely
		consult for snuffles							
		routine basis	48/49	35/43	10.97	6	-	0.01¶	
			(98%)	(81.4%)	[1.28–				
					244.62]				
		urgent basis	48/49	39/43	4.92	14	-	0.18¶	
			(98%)	(90.7%)	[0.48–				
					120.59]				

*cluster randomised controlled trial;

**pre/post design: intervention = post; control = pre;

***non-randomised controlled trial;

¶ using Fisher's Exact Test; ED: emergency department; GP: general practitioner; mo: month; Min: minimum; NNT: number needed to treat; OR: odds ratio; yr: years. Italicized p-values were those reported in original study.

**Table 2:** Effectiveness of interventions to change parent consulting rate for respiratory tract infections in children.

Study	Age	Outcome	Intervention	Control	OR	NNT	Mean	Significance	Risk
					[95%		difference		of
					CI]				bias
Francis*	6 mo-	% reconsulting by	33/256	44/272	0.77	30	-	0.34	Min.
	4 yr	2 wk follow-up	(12.9%)	(16.2%)	[0.46–				
					1.28]				
Isaacman***	<3 yr	% reconsulting to	Verbal: 1/41	8/78	0.22	13	-	0.16¶ (vs.	Likely
		PED by 3 day	(2.2%)	(10.1%)	[0.01–			C)	
		follow-up			1.84]				
			Written +		0.37	16	-	0.31¶ (vs.	
			verbal: 2/49		[0.05–			C)	
			(3.8%)		2.02]				
Morrell,		Mean							Likely
Anderson §		consultations/							
		patient/yr****							
	0–4 yr	Sore throat	0.16	0.27	-	-	0.11	*NR*	
		Cough	1.08	1.20	-	-	0.12	*NR*	
		Runny/stuffy nose	0.10	0.10	-	-	0	*NR*	
	5–14 yr	Sore throat	0.19	0.23	-	-	0.04	*NR*	
		Cough	0.31	0.40	-	-	0.09	*NR*	
		Runny/stuffy nose	0.06	0.02	-	-	−0.04	*NR*	
Roberts §	≤18 yr	Consultations/	0.185	0.303	-	-	0.118	*NR*	Likely
		person/yr							
		(pre vs. post)							
		Unnecessary	0.064	0.141	-	-	0.077	*NR*	
		consultations/							
		person/yr							
Thomson	<6 mo	Infants receiving	242/467	236/468	1.06	72	-	0.72	Min.
		RTI diagnoses	(51.8%)	(50.4%)	[0.81–				
					1.38]				
		Infants receiving	161/467	126/468	1.43	13	-	0.02	
		oral antibiotics	(34.5%)	(26.9%)	[1.07–				
					1.91]				
Usherwood	2–12 yr	Consultations/							Likely
		household							
		Sore throat	32/210	65/209	0.26	6	-	<0.001	
			(15.2%)	(31.1%)	[0.16–				
					0.42]				
		Cough	90/210	116/209	0.60	8	-	0.01	
			(43%)	(56%)	[0.40–				
					0.90]				

*cluster randomised controlled trial;

***non-randomised controlled trial;

****adjusted for children at risk for part of study year;

¶ using Fisher's Exact Test;

§ no absolute numbers given; mo: month; Min: minimum; NNT: number needed to treat; NR: not reported; OR: odds ratio; PED: paediatric emergency department; RTI: respiratory tract infection; wk: weeks; yr: years. Italicized p-values were those reported in original study.

#### Knowledge related to consulting

Outcomes were mixed in the five studies that assessed change in parent knowledge in relation to consulting [19], [20], [21], [23], [24] (**Table 1**). Interventions significantly improved parents' knowledge about RTIs compared to controls in two studies [21], [23], but did not measure effects on actual consultations. Similarly, the three studies which measured future consultation intentions also found significantly increased knowledge about appropriate reasons to consult, but again their impact on actual consultations was not measured [19], [20], [24].

#### Consultation rate

Six studies assessed the effects of interventions designed to reduce the number of consultations for RTIs [19], [21], [22], [25], [26], [27] (**Table 2**). Three studies found that providing parents with informative, illustrated booklets prior to their child becoming ill resulted in lower rates of consulting for sore throat, cough, respiratory tract infection and otitis media [22], [25], [27]. Usherwood et al found that consultations per household decreased nearly 16% for sore throat (p = 0.0002) and 13% for cough (p = 0.013) [27]. In the study by Roberts, there was a relative reduction of 40% in the number of consultations per person/year for acute otitis media and other RTIs [25]. In one study, parents received books with information on multiple symptoms of respiratory infection (for example, cough, sore throat, and runny nose), yet consulting decreased significantly among all age groups only for sore throat (p<0.05) [22]. The method of randomization was not reported in two of these studies, and generalisability may be limited given they were published 20–30 years ago [22], [27]. Thomson et al provided mothers of infants in the intervention group with a guide for assessing severity of illness, and found similar numbers of infants subsequently had RTIs recorded by a clinician (52% vs. 50%, p = 0.718), however infants in the intervention group were more likely to receive prescriptions for oral antibiotics (OR 1.43 [95%CI 1.07–1.91]) [26]. Two studies assessed interventions delivered at point of consulting and designed to reduce re-consultations within a given illness episode; neither found significant differences in proportions re-consulting between intervention and control groups [19], [21]. Adverse events (hospital admission ≤2 nights) were reported in one trial and were similar among intervention and control [19]. Adverse effects were generally not assessed, however Roberts et al noted a non-significant reduction in “necessary” consultations (those for more severe symptoms of respiratory infection, defined by the authors as “symptom clusters suggesting that diagnostic testing or drug therapy might be cost-effective”) among African American and Medicaid patients receiving the intervention [25].

### 2. Interventions to influence parents' decision towards use of antibiotics

We identified 10 studies of nine interventions designed to influence antibiotic use for RTIs in children (**Table 3**, **4**, and **5**) [28], [29], [30], [31], [32], [33], [34], [35], [36], [37]. Studies involved 2,916 participants with follow-up periods ranging from 1 day to 36 weeks. The majority of interventions took place during the consultation, with only two [29], [30] designed to influence parental attitudes or knowledge before their children became ill.

**Table 3:** Effectiveness of interventions to improve parent knowledge of appropriate antibiotic use for respiratory tract infections in children.

Study	Age	Outcome	Intervention	Control	OR	NNT	Mean	Significance	Risk
					[95%CI]		difference		of
					or				bias
					difference				
Alder	1–10	Change in							Likely
	yr	parental	Communication	-	-	-	-	0.02	
		communication	AB information	-	-	-	-	0.34	
		efficacy	Interaction	-	-	-	-	0.62	
Bauchner	6 mo-	Post-test	8.04	7.82	-	-	0.22	0.31	Likely
	3 yr	adjusted							
		knowledge							
		score							
		(range 0–11)							
Croft*	<5 yr	Median							High
		knowledge							
		score (range 0–							
		9)							
		College	7	6.5	0.5	-	-	<0.01	
		graduates							
		Non-college	6	6	0		-	0.20	
		graduates							
Maor**	8 d-	Knowledge of	45.1%	36.1%	9%	-	-	0.01	Likely
	16 yr	AB treatment							
		(>50% correct							
		answers)							
Schnellinger	<18	Knowledge	I1. Pamphlet:	(8, 8, 8)	-	-	-	I1: 0.32***	Min.
	yr	score	(8, 10, 9)					I2: 0.002***	
		(range 1–10)	I2. Video:					C: 0.26***	
		(baseline vs.	(9, 10, 10)						
		following							
		intervention vs.							
		1 mo)							

*cluster randomised controlled trial;

**Pre/post design: intervention = post; control = pre;

***within-group significance; AB: antibiotics; d: days; mo: month; Min: minimum; NNT: number needed to treat; OR: odds ratio; yr: years.

**Table 4:** Effectiveness of interventions to improve parental attitudes toward appropriate antibiotic use for respiratory tract infections in children.

Study	Age	Outcome	Intervention	Control	OR [95%	NNT	Mean	Significance	Risk
					CI] or		difference		of
					difference				bias
Taylor	<24 mo	Parental attitude							Min.
		score (group mean)							
		(range 1–6; 6 =							
		“completely agree”)							
		“Too many children	5.18	4.86	-	-	0.32	0.07	
		are treated with AB							
		when not necessary”							
		“Parents should not try	5.26	4.99	-	-	0.27	0.08	
		to persuade a doctor							
		to prescribe AB”							
		“Physicians should	5.64	5.47	-	-	0.17	0.10	
		never prescribe AB							
		when they are							
		unnecessary”							
		“Overuse of AB can	5.78	5.52	-	-	0.26	0.021	
		make bacteria more							
		resistant to AB”							
		(range 1–6, 1 =			-	-			
		“completely disagree”)							
		“Giving an AB to a	1.86	2.16	-	-	0.3	0.005‡	
		child with cold							
		symptoms can prevent							
		an infection from							
		occurring”							
		“It is worth trying an	1.93	2.34	-	-	0.41	0.001‡	
		AB when my child has							
		cold symptoms							
		for 5 days”							
		“Treatment with AB is	2.61	3.47	-	-	0.86	0.001‡	
		necessary when a							
		child's nasal							
		discharge turns from							
		yellow to green in							
		color”							
		“AB help a child's cold	1.64	2.01	-	-	0.37	0.001‡	
		symptoms clear up							
		more quickly”							
		“AB are helpful in	1.52	1.87	-	-	0.35	<0.001‡	
		treating colds”							
Wheeler**	<18 yr	% of parents in							High
		agreement with:							
		“Antibiotics should be	9/126	34/114	0.18	4	-	<0.001	
		used always or mostly”	(7.1%)	(29.8%)	[0.08–				
		(for children with cold			0.42]				
		and fever)							
		“Yes, I want	18/130	51/115	0.20	3	-	<0.001	
		antibiotics”	(13.8%)	(44.3%)	[0.10–				
					0.39]				

** Pre/post design: intervention = post; control = pre;

‡ “Statistically significant P values after correcting for multiple tests”; AB: antibiotics; mo: month; Min: minimum; NNT: number needed to treat; OR: odds ratio; yr: years.

**Table 5:** Change in parental satisfaction with a ‘no prescribing’ intervention.

Study	Age	Outcome	Intervention	Control	OR [95%	NNT	Mean	Significance	Risk
					CI] or		difference		of
					difference				bias
Chao	2–12	Proportion of parents	91/100	101/106	0.50	23	-	0.345	Min.
	yr	reporting “very or	(91%)	(95%)	[0.14–				
		extremely satisfied”			1.74]				
McCormick	6 mo-	Parent satisfaction	44.6/52	44.6/52	-	-	0	*NS*	Min.
	2 yr	score (range 0–52)							

mo: month; Min: minimum; NNT: number needed to treat; NS: not significant; OR: odds ratio; yr: years. Italicized p-values were those reported in original study.

#### Knowledge of appropriate antibiotic use

Five of the studies were set in primary care [28], [31], [33], [34], [37] and two in emergency departments [32], [36]; the remaining interventions took place at home or in day care centres (**Table 3**). Interventions most often used video or written materials (pamphlets or handouts). Four [31], [32], [33], [34] of the five studies which measured the effects of interventions on parental knowledge of appropriate antibiotic use (using a variety of different scores) found significant increases compared to the control groups, though in one study improvement only occurred among parents with college education [30], and two studies [31], [33] had higher risk of bias due to weak study design (pre/post test) and low rates of exposure to the intervention. Three of these studies used interventions designed to engage children as well as their parents, such as cartoon-animation videos and colouring books [30], [31], [32]. Schnellinger et al found that parental knowledge increased immediately and was maintained at one month (p<0.001) following a three minute cartoon-animated video, compared to parents only given pamphlets in whom knowledge improved immediately, but significantly waned at one month (p = 0.002) [32]. One study, however, found no improvement when parents were given a video and brochure (prior to an illness episode) and instructed to view it at home as often as they preferred [29]. It is unclear if the lack of effect was due to the home setting, or to specific attributes of the video (e.g. length of video was 20 minutes).

#### Attitudes towards antibiotic use

Two studies measured effects on parental attitudes towards antibiotic use (**Table 4**). Taylor et al found that a ‘personalised’ video intervention (featuring a paediatrician from the local clinic) was more effective in changing attitudes about when not to use antibiotics for specific illnesses (e.g. nasal discharge) and less effective in changing general perspectives about antibiotics (e.g. threat of antibiotic resistance) [34]. A video intervention studied by Wheeler et al [33] demonstrated moderate success in increasing the proportion of parents with appropriate attitudes towards antibiotic use (i.e. fewer parents wanting antibiotics, OR 0.20 [0.10–0.39]); however less than 60% of surveyed parents reported exposure to the intervention at any time. Despite significant improvements in parental attitudes toward judicious use of antibiotics, neither study found changes in antibiotic prescribing rates over follow-up periods of 6 to 12 months (data not shown).

#### Satisfaction with a ‘no prescribing’ approach

Two studies [36], [37] assessed parent satisfaction with a ‘watchful waiting,’ or no prescribing approach, neither of which found significant differences in parent satisfaction between intervention and control groups (**Table 5**). In addition, McCormick et al [37] found no difference in persistence of acute otitis media symptoms at 30 days, and that more children in the control group reported adverse events (side effects of antibiotics). Treatment costs for children in the intervention group (watchful waiting) averaged $11.43 per child compared to $47.41 per child in the immediate antibiotic group. Chao et al reported no adverse events or increase in consultations related to intervention [36].

### 3. Interventions to influence antibiotic use in children with RTIs

We found six studies of five interventions that reported the effects of interventions to influence antibiotic use in children with RTIs [19], [36], [38], [39], [40], [41]; all but one assessed the effect of delayed prescribing or watchful waiting therapy for AOM, the other study [19] involved a book for parents of children with RTIs.

#### Use of antibiotic prescriptions

Each of the five interventions designed to influence antibiotic use was effective compared to control, with a combined RR 0.39 (0.29, 0.53) (not shown in table). Combining a delayed or no prescribing strategy with brief education significantly decreased antibiotic use in four studies [36], [38], [40], [41], and an interactive book [19] resulted in 19% fewer children taking antibiotics during the two weeks following consultation compared to controls (p<0.001) (**Table 6**). In pooled analyses we found significant heterogeneity (p = 0.002, I^2^ = 77%) due to one study [38] in which nearly 100% of parents in the control group reported antibiotic use. After removal of this study, parents in the intervention groups were still significantly less likely to report antibiotic use, combined RR 0.46 (0.40, 0.54) (p = 0.51, I^2^ = 0%) (**Figure S3**). Although the duration of acute otitis media symptoms was slightly shorter among children prescribed immediate antibiotics [38], [41], follow-up did not show significant differences in increased risk of relapse at either three months or one year [39]. More adverse outcomes related to antibiotic use (diarrhoea) were reported in one study among children in the control group than those receiving the intervention [41].

**Table 6:** Effectiveness of interventions to influence filling antibiotic prescription for children with respiratory tract infections.

Study	Age	Outcome	Intervention	Control	OR [95% CI]	NNT	Significance	Risk of
					or difference			bias
Reduction in number of children taking antibiotics								
Chao	2–12 yr	Number of children taking	13/100	40/106	0.25 [0.11–	4	<0.0001	Min.
		AB or re-consulting	(13%)	(37.7%)	0.52]			
		for ABx						
Francis*	6 mo-	Number of children	55/246	111/263	0.39 [0.26–	5	<0.0001	Min.
	14 yr	taking AB	(22.4%)	(42.2%)	0.59]			
Little ‡	6 mo-	Number of children	36/150	132/134	74.5% (66.2%–	1	<0.0001	Min.
	10 yr	taking AB	(24%)	(98.5%)	80.7%)			
Pshetizky	3 mo-	Number of children	18/44	32/37	0.11 [0.03–	2	<0.0001	Min.
	4 yr	taking AB	(40.9%)	(86.5%)	0.36]			
Reduction in number of parents filling antibiotic prescription								
Spiro	6 mo-	Number of parents	50/132	116/133	0.09 [0.05–	2	<0.0001	Min.
	12 yr	filling AB script	(37.9%)	(87.2%)	0.17]			

*cluster randomised controlled trial;

‡ because of the small numbers in one of the cells we calculated proportional difference; AB: antibiotic; ABx: antibiotic prescription; Min: minimum; mo: month; NNT: number needed to treat; OR: odds ratio; yr: year.

## Discussion

We systematically reviewed and synthesised the evidence for the effectiveness of interventions targeted at parents to modify consulting behaviour and antibiotic use for children with RTIs. To the best of our knowledge, this is the first systematic review of interventions to increase appropriate care-seeking behaviour in parents of children with acute respiratory tract infections. Providing educational materials to parents reduced rates of consulting by up to 40% in three studies; however all were published nearly 20 years ago and therefore may not translate to contemporary primary care [22], [25], [27]. Interventions may be more successful when delivered prior to the child's illness rather than during consultations [19], [22], [25], [27], and when focussed on specific symptoms rather than generic messages about antibiotic overuse and resistance [34]. Effects were more consistent for certain symptoms (e.g. sore throat) than others (e.g. cough), which may reflect features specific to certain RTIs (particularly duration of illness which is short for sore throat and longer for cough), or merely the studies we identified [22], [27]. Delayed or no prescribing interventions reduced rates of antibiotic use by up to half. Finally, educational materials that include cartoons and illustrations and which engage the child as well as their parents may be more successful than text-only materials; this approach was successful in several studies from different settings [22], [25], [27], [30], [31], [32].

Previous reviews [42], [43], [44], [45] have approached the problem of antibiotic overuse by assessing effectiveness of interventions to reduce clinician antibiotic prescribing. Our review, in contrast, has focussed on interventions targeted to parents or caregivers. In practice, change is needed by clinicians and parents and/or patients to reduce antibiotic use and control resistance. Our finding that framing education around specific presenting symptoms may be more meaningful to parents than less focussed approaches echoes a review by Glascoe [46] of general health education interventions directed toward parents. As with reviews by Arroll [47] and Spurling [48] which assessed the effect of delayed prescriptions in patients of all ages, we also found this strategy reduces antibiotic use in children as reported by their parents; importantly, our review demonstrates that parents accept this approach.

Our review adds to the literature by integrating the research on parental consulting and antibiotic use; we feel it is crucial to consider these elements together as they influence one another, often as part of a ‘vicious cycle’ of consulting and antibiotic-seeking. Decisions to consult or use antibiotics are not isolated events. Rather, they involve interactions between several stakeholders (e.g. parents, clinicians), in multiple situations (e.g. home, GP surgery), and at multiple moments in time (RTIs occur repeatedly). Although it increased the complexity of the review, the main strength of our approach is that it allowed us to identify overarching intervention components that appear effective.

### Limitations

Our literature search was limited to studies reported in peer-reviewed journals, and therefore we may have missed relevant, unpublished research. To counter publication bias we searched multiple databases and did not limit by language. This review was focussed on studies from OECD countries (and mostly the US), which may limit generalisability to other countries or clinical settings. We included studies which used a range of study designs and follow-up periods, which limited our ability to synthesise results quantitatively. Several of the studies which showed reductions in consultation were published over 20 years ago [22], [25], [27], when prescribing for RTIs was higher generally, and thus may not apply to contemporary practice where antibiotic use has declined. Of the 20 interventions included in the review, 13 studies reported receipt of funding or support. The majority of funding organizations were governmental (only three studies [32], [33], [34] reported sole funding from private organizations). Included studies generally lacked explicit diagnostic criteria and excluded children with severe cases of infection or those at higher risk of complication. Thus, our findings may be less generalisable to these populations. Finally, few studies reported harms of interventions (e.g. missed treatment of serious infection).

### Conclusions

The key finding of our review for clinicians is that interventions (such as written materials with focussed information for parents) can reduce the number of consultations for RTIs by 10 to 40%, and that use of antibiotics can be reduced by up to half through delayed prescribing. Importantly, reductions in antibiotic use do not seem to occur at the expense of parent satisfaction. Given the high frequency of paediatric consultations in primary care, a change in parental consulting behaviour for RTIs could potentially create a ‘virtuous cycle’ of reductions in workload and antibiotic use. We found moderate evidence that interventions are more effective when delivered to parents and children. Moreover, our findings have important implications for the content, format, and timing of delivery of patient information materials (see **Table 7**). Written information with focussed content (to a specific symptom) appears to be more effective than generic messages about avoiding antibiotics or antibiotic resistance. Furthermore, altering consultations for certain RTIs (e.g. sore throat) seems to be more achievable than for others (e.g. cough). It is unclear whether this simply reflects the studies included in this review, or whether effectiveness differs for different RTIs because of parent (and clinician) perceptions about severity or risk of complications of different RTIs. Clinicians might want to look carefully at the format of the information they hand to parents; those with cartoons or illustrations seemed to be more effective than bland text. The few studies which examined video interventions showed mixed results, and none explored online technologies.

**Table 7:** Implications of findings.

Outcome	Implications for clinical practice and future research	Level of evidence
Parental knowledge related to consulting	▪ Change in knowledge was equivocal; unclear meaning of parental intent to consult due to hypothetical nature of the outcome	Weak
Parental knowledge or attitudes related to antibiotic use	▪ Cartoon-illustrated materials engage children and parents▪ Information specific to RTI symptoms, rather than general antibiotic use, may be more meaningful to parents	Moderate
Parental consulting	▪ Providing parents with written information (with cartoons and/or illustrations) reduced consulting compared to control▪ Consulting for certain RTI (e.g. sore throat) may be easier to modify than consulting for other symptoms (e.g. cough)	Moderate
Filling antibiotic prescription	▪ ‘Delayed or no prescribing’ approach with supporting educational material reduced antibiotic use without diminishing parental satisfaction	Strong

Reducing unnecessary antibiotic use in primary care with the explicit goal of avoiding further spread of antibiotic resistance is a policy priority in many countries. To some extent it has been successful – prescribing of antibiotics fell by 24% in the UK during the 1990 s [1]. Although some clinicians worry that reductions in prescribing have gone “too far” leading to increased incidence of complications of RTIs [49], [50], there is broad agreement that prescribing rates remain inappropriately high for many RTIs. Our findings provide policy makers with evidence they need to implement or commission effective interventions in community settings to reduce consultations and antibiotic use. Moreover, interventions to reduce antibiotic use do not seem to occur at the expense of parent satisfaction, although evidence for conditions other than acute otitis media was limited. GPs implementing any of the strategies identified in this review should be mindful of the possibility of unintended adverse effects, such as reducing consultations for illness episodes that ought to be managed by a GP, as seen in the Roberts study [25]. Paradoxically, reducing the overall numbers of consultations for RTIs may filter a higher proportion of children with more severe illness presenting to primary care, highlighting the need for effective strategies to identify children at highest risk of complications and who are most likely to benefit from antibiotics; our group is currently conducting such research (see http://www.targetstudy.org.uk/).

### Recommendations for future research

We have several suggestions to improve the primary research in this area. Firstly, reporting should include detailed descriptions of the intervention, the extent of exposure to the intervention, and whether the trial was conducted in communities where public media campaigns related to antibiotics were occurring (see study [29]). Secondly, follow-up periods need to be long enough to measure the longevity of interventions given that RTIs recur frequently. Further, potential outcomes should include number of symptomatic days, hospitalisations, and time off work or school [42], [51]; most importantly, adverse outcomes should be assessed and reported. Finally, we urge the use of “head-to-head trials” [42] that compare the effectiveness of several delivery formats [32] (including online resources) for communicating information to parents. Going forward, interventions should be developed to influence consulting and antibiotic use for RTIs in children, rather than address these outcomes in isolation.

RTIs in children are a common cause for consultation and antibiotic use. We found several intervention strategies effective at improving parent knowledge about RTIs and when to consult, decreasing actual number of consultations, and reducing antibiotic use. Implementing one or a combination of the approaches identified in this review may reduce unnecessary consulting and use of antibiotics for children with RTIs in primary care.

## Supporting Information

Figure S1 **Conceptual framework for consulting and prescribing antibiotics for children with respiratory tract infections: ‘virtuous’ cycle.**
(TIF)

Figure S2 **Flow of included studies.**
(TIF)

Figure S3 **Effects of interventions to influence filling antibiotic prescription for children with respiratory tract infection.**
(TIF)

Table S1 Search strategies.
(DOC)

Table S2 Quality assessment of included studies.
(DOC)

Table S3 Characteristics of included studies.
(DOC)

Checklist S1 (DOC)
