# Supplementary figures and images for: Interventions to Influence Consulting and Antibiotic Use for Acute Respiratory Tract Infections in Children: A Systematic Review and Meta-Analysis
Source: PLoS One. 2012 Jan 27;7(1):e30334. doi: 10.1371/journal.pone.0030334 (PMC3267713; doi:10.1371/journal.pone.0030334)

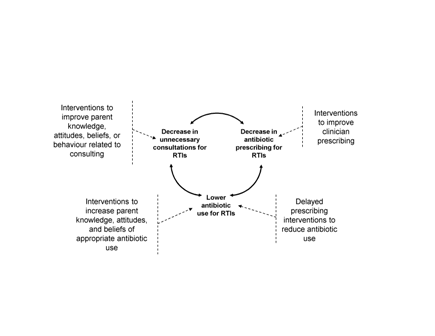

Supplement: Figure S1 — Conceptual framework for consulting and prescribing antibiotics for children with respiratory tract infections: ‘virtuous’ cycle. (TIF) [file pone.0030334.s001.tif]

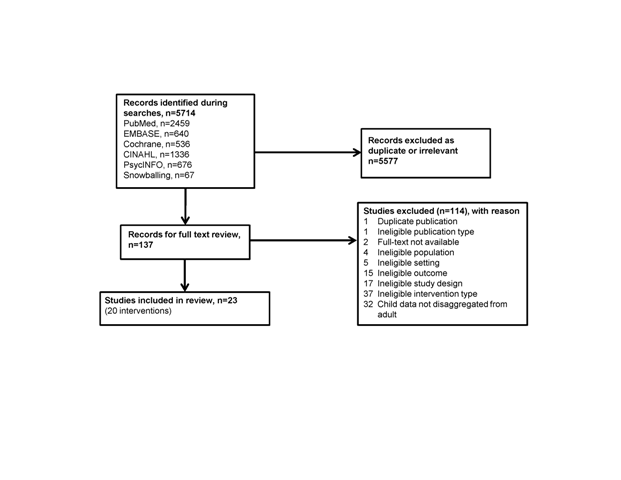

Supplement: Figure S2 — Flow of included studies. (TIF) [file pone.0030334.s002.tif]

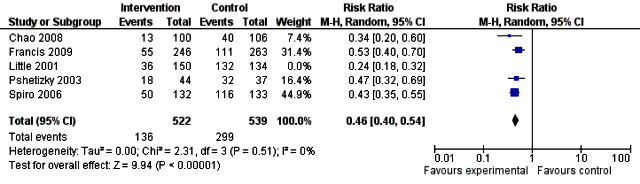

Supplement: Figure S3 — Effects of interventions to influence filling antibiotic prescription for children with respiratory tract infection. (TIF) [file pone.0030334.s003.tif]
