# Supplementary material for: Interventions to Influence Consulting and Antibiotic Use for Acute Respiratory Tract Infections in Children: A Systematic Review and Meta-Analysis
Source: PLoS One. 2012 Jan 27;7(1):e30334. doi: 10.1371/journal.pone.0030334 (PMC3267713; doi:10.1371/journal.pone.0030334)
Supplement: Table S1 — Search strategies. (DOC) [file pone.0030334.s004.doc]

**Table S1: Database search strategies**

PubMed search strategy

| #1 | respiratory tract infection[MeSH Terms] OR respiratory infection* OR rti OR lrti OR urti OR lri OR uri OR chest infection* OR cough OR (sore throat) OR pharyngitis* OR nasopharyngitis* OR laryngitis* OR earache* OR "ear ache*" OR ear infection* OR "glue ear" OR "ear disease*" OR otalgia* OR otorrhoea* OR otorrhea* OR "hearing loss" OR (otitis media) OR bronchitis* OR bronchiolitis* OR croup* OR tracheitis* OR sinusitis* OR (common cold) OR rhinosinusitis* OR rhinitis* OR tonsillopharyngitis* OR tonsillitis* OR dyspnoea OR congestion OR (lung consolidation) OR (lobar pneumonia) OR (difficult breath*) OR respiration disorder* OR "minor illness" OR self-limiting OR "self limiting" OR "acute illness" |
| --- | --- |
| #2 | child* OR schoolchild* OR preschool* OR pediatric* OR paediatric* OR parent OR parents OR parental OR mother OR father OR mom OR dad OR mum OR caregiver OR guardian OR carer OR infant OR infancy |
| #3 | intervention* OR implementation OR dissemination OR strategy OR education* OR information* OR communication* OR program* OR interactive OR decision aid OR decision* OR advice OR advise OR guidance OR train OR training* OR leaflet* OR book* OR booklet* OR brochure* OR video* OR cd OR cd-rom OR cdrom OR handout* OR website OR outreach* OR poster OR posters OR pamphlet* OR guideline* OR reminder* OR (audit and feedback) OR marketing OR printed material OR computer based OR (clinical reminder system) OR (local opinion leader) OR (decision support system) OR incentive OR multifaceted |
| #4 | office visit OR consult* OR reconsult* OR re-consult* OR attendance OR attender OR health seek* OR help seek* OR care seek* OR visit OR management OR utilize* OR repeat* AND consult* OR consultat* OR re-visit* OR house calls[MeSH Terms] OR referral and consultation[MeSH Terms] OR antibiotic* |
| #5 | tuberculosis[Title/Abstract] OR asthma[Title/Abstract] OR cystic fibrosis[Title/Abstract] OR HIV[Title/Abstract] |
| #6 | ((((#1) AND #2) AND #3) AND #4) NOT #5 |

EMBASE search strategy

| #1 | 'respiratory tract infection'/exp |
| --- | --- |
| #2 | respiratory AND infection OR respiratory AND infections OR rti OR irti OR urti OR iri OR uri OR chest AND infection OR chest AND infections OR cough OR (sore AND throat) OR pharyngitis* OR nasopharyngitis* OR laryngitis* OR earache* OR 'ear ache' OR ear AND aches OR ear AND infection* OR 'glue ear' OR 'ear disease' OR 'ear diseases' OR otalgia* OR otorrhoea* OR otorrhea* OR 'hearing loss' OR (otitis AND media) OR bronchitis* OR bronchiolitis* OR croup* OR tracheitis* OR sinusitis* OR (common AND cold) OR rhinosinusitis* OR rhinitis* OR tonsillopharyngitis* OR tonsillitis* OR dyspnoea OR congestion OR (lung AND consolidation) OR (lobar AND pneumonia) OR (difficult AND breath*) OR respiration AND disorder* OR 'minor illness' OR 'self limiting' OR 'acute illness' AND [embase]/lim |
| #3 | #1 OR #2 |
| #4 | child* OR schoolchild* OR preschool* OR pediatric* OR paediatric* OR parent OR parents OR parental OR mother OR father OR mom OR dad OR mum OR caregiver OR guardian OR carer OR infant OR infancy AND [embase]/lim |
| #5 | intervention* OR implementation OR dissemination OR strategy OR education* OR information* OR communication* OR program* OR interactive OR decision AND aid OR decision* OR advice OR advise OR guidance OR train OR training* OR leaflet* OR book* OR booklet* OR brochure* OR video* OR cd OR 'cd rom' OR cdrom OR handout* OR website OR outreach* OR poster OR posters OR pamphlet* OR guideline* OR reminder* OR (audit AND feedback) OR marketing OR printed AND material OR computer AND based OR (clinical AND reminder AND system) OR (local AND opinion AND leader) OR (decision AND support AND system) OR incentive OR multifaceted AND [embase]/lim |
| #6 | office AND visit OR consult* OR reconsult* OR attendance AND [embase]/lim |
| #7 | attender OR health AND seek* OR help AND seek* OR care AND seek* OR visit OR management AND [embase]/lim |
| #8 | utilize* OR repeat* AND consult* OR consultat* OR revisit* OR house AND calls AND [embase]/lim |
| #9 | referral AND consulation OR antibiotic* AND [embase]/lim |
| #10 | #6 OR #7 OR #8 OR #9 |
| #11 | tuberculosis:ab,ti AND [embase]/lim |
| #12 | asthma:ab,ti AND [embase]/lim |
| #13 | cystic AND fibrosis:ab,ti AND [embase]/lim |
| #14 | hiv:ab,ti AND [embase]/lim |
| #15 | #11 OR #12 OR #13 OR #14 |
| #16 | #3 AND #4 AND #5 AND #10 |
| #17 | #16 NOT #15 |

CINAHL search strategy

| S1 | (MH "Tuberculosis+") |
| --- | --- |
| S2 | (MH "Human Immunodeficiency Virus+") |
| S3 | (MH "Cystic Fibrosis") |
| S4 | (MH "Asthma+") |
| S5 | S1 or S2 or S3 or S4 |
| S6 | (MH "Child+") OR (MH "Infant+") |
| S7 | (MH "Respiratory Tract Infections+") |
| S8 | (MH "Office Visits") |
| S9 | (MH "Patient Education") |
| S10 | (MH "Antibiotics+") |
| S11 | (MH "Prescribing Patterns") |
| S12 | (MH "Behavior and Behavior Mechanisms+") |
| S13 | S8 or S9 or S10 or S11 or S12 |
| S14 | S6 and S7 and S13 |
| S15 | S14 not S5 |

PsycINFO search strategy

| 1 | exp Respiratory Tract Disorders/ |
| --- | --- |
| 2 | (child* or infant* or schoolchild* or preschool* or pre-school* or toddler*).mp. [mp=title, abstract, heading word, table of contents, key concepts] |
| 3 | exp Tuberculosis/ |
| 4 | exp HIV/ |
| 5 | exp Cystic Fibrosis/ |
| 6 | exp Asthma/ |
| 7 | 3 or 4 or 5 or 6 |
| 8 | 1 and 2 |
| 9 | 8 not 7 |

Cochrane Library search strategy

| #1 | (respiratory tract infection*) OR (respiratory infection*) OR (rti) OR (lrti) OR (urti) OR (lri) OR (uri) OR (chest infection*) OR (cough) OR (sore throat) OR (pharyngitis*) OR (nasopharyngitis*) OR (laryngitis*) OR (earache*) OR (ear ache*) OR (ear infection*) OR (glue ear) OR (ear disease*) OR (otalgia*) OR (otorrhoea*) OR (otorrhea*) OR (hearing loss) OR (otitis media) OR (bronchitis*) OR (bronchiolitis*) OR (croup*) OR (tracheitis*) OR (sinusitis*) OR (common cold) OR (rhinosinusitis*) OR (rhinitis*) OR (tonsillopharyngitis*) OR (tonsillitis*) OR (dyspnoea) OR (congestion) OR (lung consolidation) OR (lobar pneumonia) OR (difficult breath*) OR (respiration disorder*) OR (minor illness) OR (self-limiting) OR (self limiting) OR (acute illness) in Title, Abstract or Keywords |
| --- | --- |
| #2 | (child*) OR (schoolchild*) OR (preschool*) OR (pediatric*) OR (paediatric*) OR (parent) OR (parents) OR (parental) OR (mother) OR (father) OR (mom) OR (dad) OR (mum) OR (caregiver) OR (guardian) OR (carer) OR (infant) OR (infancy) in Title, Abstract or Keywords |
| #3 | (intervention*) OR (implementation) OR (dissemination) OR (strategy) OR (education*) OR (information*) OR (communication*) OR (program*) OR (interactive) OR (decision aid) OR (decision*) OR (advice) OR (advise) OR (guidance) OR (train) OR (training*) OR (leaflet*) OR (book*) OR (booklet*) OR (brochure*) OR (video*) OR (cd) OR (cd-rom) OR (cdrom) OR (handout*) OR (website) OR (outreach*) OR (poster) OR (posters) OR (pamphlet*) OR (guideline*) OR (reminder*) OR (audit and feedback) OR (marketing) OR (printed material) OR (computer based) OR (clinical reminder system) OR (local opinion leader) OR (decision support system) OR (incentive) OR (multifaceted) in Title, Abstract or Keywords |
| #4 | (office visit) OR (consult*) OR (reconsult*) OR (re-consult*) OR (attendance) OR (attender) OR (health seek*) OR (help seek*) OR (care seek*) OR (visit) OR (management) OR (utilize*) OR (repeat*) AND (consult*) OR (consultat*) OR (re-visit*) OR (house calls) OR (referral and consultation) OR (antibiotic*) in Title, Abstract or Keywords |
| #5 | (tuberculosis) OR (asthma) OR (cystic fibrosis) OR (HIV) in Title, Abstract or Keywords in Cochrane Database of Systematic Reviews |
| #6 | ((((#1) AND #2) AND #3) AND #4) NOT #5 |
