# Supplementary material for: Interventions to Influence Consulting and Antibiotic Use for Acute Respiratory Tract Infections in Children: A Systematic Review and Meta-Analysis
Source: PLoS One. 2012 Jan 27;7(1):e30334. doi: 10.1371/journal.pone.0030334 (PMC3267713; doi:10.1371/journal.pone.0030334)
Supplement: Table S2 — Quality assessment of included studies. (DOC) [file pone.0030334.s005.doc]

**Table S2: Quality assessment of included studies[[1]](#footnote-2)**

| **Author** | **Design** | **Randomization / comparability of groups** | **Blinding** | **Intervention description** | **Exposure to intervention** | **Generalisability** | **Risk of bias** |
| --- | --- | --- | --- | --- | --- | --- | --- |
| **Alder, 2005** | RCT | Unclear – method not reported | Unclear – not reported | Low – intervention described | Unclear – not reported | Low – US primary care setting | Likely |
| **Bauchner, 2001** | RCT | Unclear – method not reported | Unclear – not reported | Low – intervention described | Unclear – parents reported watching video; exposure not standardized and no way to validate parental report | Unclear – US, study limited to parents with video player at home, unclear which parent characteristics were associated w/ or w/o video player | Likely |
| **Chao, 2008** | RCT | Low – method reported | Low – blinding of outcome assessor | Low – intervention described | Low – exposure occurred in consultation | High – US, ED setting, limited to non-severe AOM cases | Minimum |
| **Croft, 2007** | CRCT | High – method not described, baseline differences between groups | Unclear – not reported | Unclear – presentations to day care centres were script-based but varied in practice. Unclear how parent information was distributed | High – less than 50% of parents were exposed to intervention | High – US, day care setting | High |
| **Francis, 2009** | CRCT | Low – method reported | High – clinicians aware of group assignment | Low – intervention described, booklet available online | Low – exposure took place during consultation | Low – UK primary care patient population | Minimum |
| **Herman, 2009** | P/P | NA | NA | Low – intervention described | Low – exposure occurred in consultation | High – US ED setting, primarily low-income Hispanic population | Likely |
| **Isaacman, 1992** | NRCT | Low – authors report there were no significant differences between groups | NA | Low – intervention described | Low – exposure took place during consultation | High – US, ED setting, 19 year old study | Likely |
| **Little, 2001** | RCT | Low – method reported | High – open trial | Low – standardized advice used and described | Low – exposure occurred in consultation | Low – GPs in the UK, diagnosis reflected regular practice | Minimum |
| **Maor, 2010** | P/P | NA | NA | Low – intervention described | High – 57.4% of respondents reported exposure | Low – general practice, Israel | Likely |
| **McCormick, 2005** | RCT | Low – method reported | Low – blinding of investigators | Low – intervention described, standardized | Low – exposure occurred in consultation, all participants received assigned intervention as reported in study | Unclear – US academic setting, limited to cases of non-severe AOM | Minimum |
| **Morrell/ Anderson, 1980** | RCT | Unclear – method not reported | Unclear – not reported | Low – standard booklet, adequately described | Low - % parents using booklet is reported | High – 30 year old study | Likely |
| **Pshetizky, 2003** | RCT | Low – method reported | Low – blinding of outcome assessor | Low – intervention described | Low – exposure occurred during consultation^18^ | Unclear – primary care clinics in Israel, but limited to non-severe AOM cases | Minimum |
| **Robbins, 2003** | RCT | Unclear – method not reported | Low – blinding of outcome assessor | High – booklet length unclear, unclear what illnesses are included in book | Low - % receiving home visit component of intervention reported in study | High – small rural setting | Likely |
| **Roberts, 1983** | RCT | Low – method reported | Low – blinding of outcome assessors | Low – intervention described | Unclear – not reported | High –28 year old study, US | Likely |
| **Schnellinger, 2010** | RCT | Low – method reported | Unclear – not reported | Unclear – content unclear, other than information on ‘appropriate antibiotic use’ | Low – exposure occurred at consultation | High – US, ED setting | Minimum |
| **Spiro, 2006** | RCT | Low – method reported | Low – blinding of outcome assessor | Low – intervention described | Low – exposure occurred at consultation | High – US, ED setting, limited to children with non-severe AOM and with access to health care | Minimum |
| **Taylor, 2003** | RCT | Low – method reported in follow-up study | Unclear – not reported | Low – intervention described | Unclear – not reported | Low – US, research-based practice centre | Minimum |
| **Thomson, 1999** | RCT | Low – method reported | Low – practice staff were not informed of group assignment | Low – standard illness scoring tool | Unclear – no data on receipt | Low –UK GP setting | Minimum |
| **Usherwood, 1991** | RCT | Unclear – method not reported | Unclear – not reported | Low – intervention described | Low - % of parental receipt reported | High – 20 year old study | Likely |
| **Wheeler, 2001** | P/P | NA | NA | Low – intervention described | High – reported exposure low, anecdotal evidence that video would get turned off as study period progressed | Unclear – US paediatric clinic setting, limited data on patient and provider characteristics | High |

CRCT: Cluster randomised controlled trial; NA: criterion not applicable for study design; NRCT: Non-randomised controlled trial; P/P: One group pre/post test; RCT: Randomised controlled trial.

1. Each study receives a judgment of “low” “high” or “unclear” risk of bias for each criterion. Quality score is based on overall uncertainty or evidence of bias, resulting in a judgement of “minimum” “likely” or “high” risk of bias for each study. [↑](#footnote-ref-2)
