# Supplementary material for: Interventions to Influence Consulting and Antibiotic Use for Acute Respiratory Tract Infections in Children: A Systematic Review and Meta-Analysis
Source: PLoS One. 2012 Jan 27;7(1):e30334. doi: 10.1371/journal.pone.0030334 (PMC3267713; doi:10.1371/journal.pone.0030334)
Supplement: Table S3 — Characteristics of included studies. (DOC) [file pone.0030334.s006.doc]

**Table S3: Characteristics of included studies**

| **Study** | **Design** | **Setting** | **Participants** | **Children** | **Intervention** | **Comparison** | **Follow-up** | **Outcome** | **Measure** |
| --- | --- | --- | --- | --- | --- | --- | --- | --- | --- |
| **Alder**  **2005**  **USA** | RCT | GP | Parents  I1 = 20  I2 = 20  I3 = 20  C = 20 | 1-10 yr, with earache, sore throat, cough, congestion, or fever | I1: Parents practiced asking and answering four questions in role-playing exercises (“what is causing my child’s illness, when should I expect my child to feel better, when should I call your office back if my child is not feeling better, what can I do to help my child feel better at home?”) and discussed clinician characteristics that hinder communication. Parents taught breathing techniques to use at beginning of consultation.  I2: Parents given pamphlet and fact sheet with information on AB, bacteria and viruses, AB resistance, proper use.  I3: Combination of I1 and I2.  (all interventions lasted ~ 7 min) | Nutrition education (7 min) | <1 day | Parent attitude, self-efficacy | Questionnaire |
| **Bauchner**  **2001**  **USA** | RCT | Home | Parents  I = 103  C = 103 | 6 mo-3 yr | Parents given 20 minute video to watch at home as often as they preferred. Content on antibiotic resistance, common childhood illness, difference between bacterial and viral infections regarding AB treatment, importance of adherence to AB treatment, and causes of AB resistance; video included parent-clinician encounters, graphics, and education. Video development included parent surveys and focus groups. Parents also received educational brochure. | No intervention | 2 mo | Parent knowledge, belief, behaviour | 11 item true/false questionnaire; agreement with 5 statements (likert scale); adherence to 5 prescribed practices (likert scale) |
| **Chao**  **2008**  **USA** | RCT | PED (under-served urban area) | Parents | 2-12 yr with acute otitis media  I =117  (median 5 yr)  C = 115  (median 3.73 yr) | Observation - parents told to reconsult if child’s symptoms did not improve in 2-3 days (no ABx given at initial consultation). Parents also received discharge instruction sheet and ibuprofen or acetaminophen and/or antipyrine + benzocaine ear drops. | Observation therapy with prescription (instructed to wait 2-3 days before filling to see if symptoms resolve) + discharge instruction sheet + pain medication | 3 days | Parent satisfaction | Standardized structured survey |
| **Croft**  **2007**  **USA** | CRCT | DC | Parents  I = 298  C = 361 | < 5 yr | Day care staff given presentation on bacterial and viral infection, infection control methods, AB resistance, and appropriate AB use, and were instructed to distribute brochure, poster, stickers, colouring sheets to parents. | Parents received surveys but did not receive information or educational materials from day care staff | ~30 days | Parent knowledge and attitude | Parent survey |
| **Francis**  **2009**  **UK** | CRCT | GP | Practices  n = 61  Parents  Clinicians | 6 mo–14 yr  with respiratory tract infection lasting less than 7 day  I = 274  (mean 5.1 yr)  C = 284  (mean 5.3 yr) | 8-page interactive book given to parents and used during consultation with clinician to foster discussion of parental concerns, expectations, and to explain symptom course, treatment, and need for reconsultation. Booklet included information on lack of effect of AB on symptom duration, side effects, allergic reactions, resistance. | Usual care | 2 wk | Proportion of children who reconsulted for RTI over 2 wk period; intent to consult for similar illness in future | Phone interview |
| **Herman**  **2009**  **USA** | P/P | PED | Parents  Pre = 113  Post = 61 | < 18 yr, median age 48 mo, presenting with non-urgent complaint | Parents received *What to Do When Your Child Gets Sick*, a book with information on common childhood illnesses, and verbal instruction (5-10 minutes) on how to use it. | (no control group) | 6 mo | % of parents who would/would not consult | Phone interview |
| **Isaacman**  **1992**  **USA** | NRCT | PED | Parents  I1 = 52  I2 = 61  C = 84 | I1 = 26.9 mo  I2 = 26.4 mo  C = 25.2 mo  (mean age), with otitis media | I1:Standardized discharge instructions (verbal) delivered during consultation  I2: Standardized discharge instructions (written and verbal) delivered during consultation  (Instructions included information on otitis media, treatment, signs of improvement, and signs to reconsult). | Usual care (verbal or handwritten discharge instructions provided during consultation) | 3 days | Proportion of children who reconsulted over 3 day period; parental knowledge | Phone interview |
| **Little**  **2001**  **UK** | RCT | GP | Parents | 6 mo-10 yr with acute otitis media,  I = 164  C = 151 | Delayed prescription (prescription available at GP surgery). Parents advised to wait and fill Rx if symptoms not resolved at 72 hours. Also received standardized advice on self-management (use of paracetamol) and the disadvantages of AB use (side effects, resistance). | Immediate antibiotic prescription and standardized advice that taking antibiotics may lessen symptoms and prevent complications; parents also advised to take full AB course and to give children paracetamol for pain relief | 1 wk | Antibiotic use (parent report of using AB at some point during illness) | Symptom diary; parent response to validated likert scale; medical records/ GP notes |
| **Maor**  **2010**  **Israel** | P/P | GP | Parents  Pre = 868  Post = 688 | 8 d–16 yr | Poster in clinics and waiting rooms advocating judicious AB use, pamphlets with information on bacteria, viruses, when AB should be taken, side effects, and resistance. Clinicians were asked to give explanation if no AB were prescribed. Children given colouring book. | (Historical data prior to intervention) | 1 mo | Parent attitude and knowledge | Self-administered questionnaire |
| **McCormick**  **2005**  **USA** | RCT | UPC | Parents | 6 mo-12 yr  with acute otitis media,  I = 111  C =112 | Watchful waiting therapy, in which no antibiotics are prescribed + brief verbal education on definition and causes of ear infection, characteristics of severe and non-severe acute otitis media, AB resistance, costs of AB, rate of symptom response to AB, and possible adverse outcomes of immediate vs. watchful waiting treatment (5-10 minute) + thermometer and saline nose drops and/or cerumen removal drops, ibuprofen and decongestant/antihistamine. | Immediate antibiotic prescription + verbal education on definition and causes of ear infection, characteristics of severe and non-severe acute otitis media, resistance, costs of AB, rate of symptom response to AB, possible adverse outcomes of immediate vs. watchful waiting treatment (5-10 minute) + thermometer and saline nose drops and/or cerumen removal drops, ibuprofen and decongestant/ antihistamine | 1 mo | Parent satisfaction | Parent questionnaire, adapted from pre-existing instrument |
| **Morrell/ Anderson**  **1980**  **UK** | RCT | Home | Family members (284 families)  I= 521  C = 478 | < 15 yr | 16-page cartoon-illustrated book. Information on symptoms, appropriate medication, and when to consult (sore throat, cough, runny/ stuffy nose, vomiting, diarrhoea, minor trauma). | No intervention | 12 mo | Mean number of consultations per patient year | Medical record review |
| **Pshetizky**  **2003**  **Israel** | RCT | GP | Parents | 3 mo-4 yr, with acute otitis media,  I = 44  n = 37 | AB prescription with instruction to collect if symptoms no better in 48 hours + brief verbal education on acute otitis media (complications may occur with or without AB treatment; advice to use paracetamol for fever or analgesia). | AB prescription (no education) | 1 wk | Antibiotic use (parent report of giving their child AB) | Phone interview |
| **Robbins**  **2003**  **UK** | RCT | Home | Parents  I = 60  C = 60 | Infants  aged 6 wk | *Caring for Kids* book and nurse home visit. Information on parent’s experience of child illness, common illnesses, home care, and how to contact GP. | Usual care (standard nurse home visit) | 6 mo | Knowledge related to caring for child | Self-administered questionnaire |
| **Roberts**  **1983**  **USA** | RCT | GP | Families  I = 433  C = 444 | < 18 yr | 4-page pamphlet (with cartoon illustrations), patient reminder, thermometer and brief verbal education (5 min) Content covered when to consult and advice on self-management; pamphlet included space to record symptoms. | Usual care | 11 – 17 mo | Consultations per person per year for respiratory illness and otitis media | Medical record review |
| **Schnellinger**  **2010**  **USA** | RCT | PED (urban) | Parents  I1 = 79  I2 = 83  C= 84 | < 18 yr  (majority were under 11 yr) | I1: Parents were given pamphlet on  on appropriate AB use and AB resistance to review for 15 minutes (pamphlet was then returned to research assistant).  I2: Parents viewed a 3 minute animated video one time during consultation. Content on appropriate AB use and AB resistance. | No intervention | 1 mo | Parent knowledge | Knowledge survey |
| **Spiro**  **2006**  **USA** | RCT | PED | Parents | 6 mo-12 yr, with acute otitis media,  I = 138  (median 3.6 yr)  n = 145  (median 3.2 yr) | Parents given ‘wait and see prescription’ and written and verbal instructions not to ABx fill unless symptoms worsened or had not resolved at 48 hr; also given ibuprofen and ear drops for symptom management. (ABx expired at 3 days). | Immediate prescription, and written and verbal instructions to fill and use ABx immediately; ibuprofen and ear drops for symptom management | 4-6 days | Antibiotic use (parent report of filling ABx) | Telephone interview |
| **Taylor**  **2003**  **USA** | RCT | GP | Parents  I = 252  C = 247 | < 24 mo | Parents were given a 5-minute personalized videotape message featuring a paediatrician from the local clinic; pamphlet on judicious use of AB; and instruction to review material and discuss any questions with child’s clinician (clinicians were blinded to parent groups).  Pamphlet and questionnaire mailed to parents 6 wks after enrolment. | 3 pamphlets on injury prevention (each focusing on different age group); instruction to review material and discuss questions with child’s clinician. Pamphlets and questionnaire mailed to parents 6 wks after enrolment | 6 wk | Parent attitude | Self-administered questionnaire |
| **Thomson**  **1999**  **UK** | RCT | Home | Mothers  I = 497  C= 500 | Infants  < 6 mo | *Baby Check*, an illustrated illness scoring system + accident prevention leaflet. | Accident prevention leaflet | 6 mo | Infants receiving RTI diagnoses and  oral ABx | Medical record review |
| **Usherwood**  **1991**  **UK** | RCT | Home | Families  I = 210  C = 209 | 2–12 yr | 8-page cartoon-illustrated booklet. Information on symptoms, when to consult, home care advice for cough, fever, sore throat, diarrhoea, vomiting. | No intervention | 12 mo | Total frequencies of consultations per household per year | Medical record review |
| **Wheeler**  **2001**  **USA** | P/P | GP | Parents  n = 771 | < 18 yr, median 3 yr | 8 minute video on judicious use of AB. Video featured expert as well as local clinicians, nurses, and families, and was based on pamphlet (video shown in waiting room, pamphlets were also available in waiting room but were not given directly to parents). | 20 minute cartoon video on stimulant drug abuse (shown in waiting room during baseline period and interspersed throughout study) | 36 wk | Parent attitude | Questionnaire,  Chart review |

AB: Antibiotic; ABx: Antibiotic prescription; C: Control; CRCT: Cluster randomised controlled trial; DC: Day care centre; GP: General practice; I: Intervention; IL: Israel; min: minutes; mo: months; NRCT: Non-randomised controlled trial; PED: Paediatric emergency department; P/P: One group pre/post test; RCT: Randomised controlled trial; RTI: Respiratory tract infection; UK: United Kingdom; UPC: University paediatric clinic; USA: United States; w: weeks; yr: years.
